# *Bushen Jianpi Quyu* Formula Alleviates Myelosuppression of an Immune-Mediated Aplastic Anemia Mouse Model via Inhibiting Expression of the PI3K/AKT/NF-*κ*B Signaling Pathway

**DOI:** 10.1155/2022/9033297

**Published:** 2022-04-14

**Authors:** Hangchao Li, Lina Ji, Yingying Shen, Danqing Fu, Dijiong Wu, Baodong Ye

**Affiliations:** ^1^The First School of Clinical Medicine, Zhejiang Chinese Medical University, Hangzhou, Zhejiang, China; ^2^Department of Hematology, The First Affiliated Hospital of Zhejiang Chinese Medical University, Hangzhou, Zhejiang, China; ^3^School of Basic Medical Sciences, Zhejiang Chinese Medical University, Hangzhou, Zhejiang, China

## Abstract

*Bushen Jianpi Quyu* Formula (BSJPQYF), an experienced formula, has been used to treat aplastic anemia (AA) more than three decades. To determinate the effect of BSJPQYF on AA, we constructed an immune-mediated AA mouse model. All mice were divided into four groups: control, model, low dose (0.85 g/mL), and high dose (1.7 g/mL BSJPQYF) group. They were administered with different concentrations of BSJPQYF or normal saline for 14 days. Besides, components of BSJPQYF were analyzed by electrospray ionization and mass spectrometry (ESI-MS). Subsequently, mouse peripheral blood and femurs were collected, and bone marrow mesenchymal stem cells (BMSCs) were isolated by fluorescence-activated cell sorting (FACS). Among them, tumor necrosis factor-*α* (TNF-*α*), transforming growth factor-*β* (TGF-*β*), and interferon-*γ* (IFN-*γ*) were measured by ELISA assay, PI3K, AKT, p-AKT, NF-*κ*B, p-NF-*κ*B, TNF-*α*, and cleaved caspase-3 proteins were detected by western blot. Compared with standard compounds, we identified three compounds of BSJPQYF, namely, icariin, kaempferol and tanshinone iia, as potentially effective compounds for the treatment of AA. Through an *in vivo* study, we found the administration of BSJPQYF in high dose for 14 days could significantly increase peripheral blood count and bone marrow (BM) cells, meanwhile decrease TNF-*α*, TGF-*β*, and IFN-*γ* levels. Besides, it could suppress the protein expression of PI3K and the phosphorylation of AKT and NF-*κ*B to restrict the protein expression of TNF-*α*, eventually reduce the protein expression of cleaved caspase-3. This study demonstrated the therapeutic effects of BSJPQYF in AA, which could alleviate myelosuppression through inhibiting the expression of the PI3K/AKT/NF-*κ*B signaling pathway.

## 1. Introduction

Aplastic anemia (AA), a rare hematologic disease, presents pancytopenia [1]. The current treatment for AA includes immunosuppressive therapy, haemopoietic stem cell transplant, and eltrombopag [2–4]. Although these first-line treatments have been clinically proven effective, they are limited by side effects, absence of HLA-matched donor, and high cost. In China, the application of Chinese herbal medicines was extensively taken as the complementary therapy for AA due to its effectiveness, safety, less toxicity, and few side effects [5, 6]. *Bushen Jianpi Quyu* Formula (BSJPQYF), as an experienced formula, is used to treat AA for preventing from bleeding and inflammation, reducing adverse reactions of immunosuppressive therapy (IST), as well as delaying disease progress in our center for more than three decades [7]. We previously reported it could improve adhesion molecules expression levels of bone marrow mesenchymal stem cells (BMSCs) in AA patients [8, 9]. Besides treatment with the kidney-reinforcing, blood-activating, and stasis-removing herbals could reduce the rate of graft failure and treatment-related mortality and improve the rate of overall survival (OS) in severe AA patients with allogeneic hematopoietic stem cell transplantation (allo-HSCT) [10]. However, its underlying mechanism for AA treatment remains unclear.

Current studies mainly focus on the abnormal differentiation and proliferation of BMSCs in AA [11–15]. In this study, we aimed to determinate apoptosis of BMSCs and explore the effects of BSJPQYF in the AA mice model. In addition, we wanted to identify the components in BSJPQYF to contribute to further clinical precision therapy.

## 2. Materials, Animals, and Methods

### 2.1. Drugs

BSJPQYF was composed of 16 herbs (Table 1). All raw herbal medicines for BSJPQYF were purchased from the first affiliated hospital of Zhejiang Chinese Medical University (Hangzhou, China). The total 228 g of mixed medicine were soaked in 1000 mL distilled water for 20 min before boiled for 60 min, then boiled for another 60 min after the residue extracted. The filtrate was collected and concentrated to 1.7 g crude drug/mL, then preserved at 4°C, and re-warmed before administration.

### 2.2. Electrospray Ionization and Mass Spectrometry (ESI-MS) Method for BSJPQYF Component Analysis

Icariin (HPLC ≥98%), kaempferol (HPLC ≥98%), and tanshinone iia (HPLC ≥98%) were prepared (Yuanye, Shanghai, China). The electrospray ionization (ESI) was used as the ionization source by a Q-TOF SYNAPT G2-Si high-definition mass spectrometer (Waters, MA, USA). The separation was performed by a Waters ACQUITY UPLC Cortecs T3 Column (2.1 × 100 mm, 1.7 *μ*m) with Cortecs T3 Van Guard (2.1 × 50 mm, 1.7 *μ*m). Brieﬂy, in ESI-MS analysis, the capillary voltage was set as 3.0 kV. The MSE continuum mode was carried out over the range of *m*/*z* 50–1500. Each sample was injected into the column in 2 *μ*L and employed with a gradient elution. The column and autosampler temperature were maintained at 35°C and 25°C. The gradient elution consisted of A (acetonitrile) and B (0.1% formic acid in water), the flow rate was controlled at 0.3 mL/min. The gradient program was set as follows: 0–2 min, 5%A; 2–30 min, 5%–50% A; 30–35 min, 50%–100% A; and 35–36 min, 100% A.

### 2.3. Immune-Mediated AA Mouse Model Construction and BSJPQYF Treatment *In Vivo*

Specific pathogen free female BALB/c mice and female DBA/2 mice at 6–8 weeks of age, weighing 20–22 g, were obtained from Slac Laboratory Animal Co., Ltd. (Shanghai, China) [16]. The experimental animal license number is SYXK (Zhejiang) 2018–0012. All procedures and animal experiments were approved by the Animal Care and Use Committee of the Animal Center of Zhejiang Traditional Chinese Medicine University (No. ZSLL-2010-66). Totally 40 mice were divided into four groups with 10 mice in each group: control group, AA model group, low dose (0.85 g/mL), and high dose (1.7 g/mL) of BSJPQYF group. The drug dosage setting is based on the BSJPQYF daily dosage of the adult 60 kg weight, which is 3.8 g crude drug/kg/day. The adult daily dose was converted into the high dosage of mouse, which is 0.684 g/kg/day [17]. Based on this dosage, the low dose and high dose of BSJPQYF filtrate was set as 0.85 g/mL and 1.7 g/mL (20 mL/kg body weight per day), respectively. The AA mouse model was established as previously described [16]. Briefly, BALB/c mice were exposed to 6.0 Gy total body irradiation (TBI) at approximately 1.0 Gy/min for 6 min. Then, it was administered per mouse with 1 × 10^6^ lymphocytes harvested from lymph glands of DBA/2 mice *via* tail vein injection within 4 h after irradiation. 2 days after the establishment of AA model, BSJPQYF with different concentrations were intragastrically administered for 14 days at 0.4 mL per day, respectively, while the mice in the control and model groups were administered with saline solution in the same volume.

### 2.4. Bone Marrow Histology

After 14 days of intervention, all mice were sacrificed by cervical dislocation. Femurs were harvested, fixed in 10% neutral buffered formalin for 24 h, decalcified with EDTA for 14 days, dehydrated with 50%–95% alcohol, then processed by xylene, embedded in paraffin, sectioned, and stained with hematoxylin and eosin (HE) for mouse bone marrow histological examination as previously described [18].

### 2.5. BMSC Isolation and Identification

**Figure 1:**
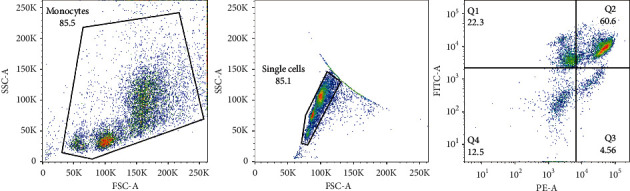
Gating strategy for cell sorting of CD45-FITC-CD44PE^+^ cells isolated from BM cells.

Femurs at both the proximal and distal ends of the bones were made small cuts carefully. Whole BM cells were extracted from the femurs by using a 25G needle to flush with a DEME medium containing 10% FBS [19]. The washes were collected from each of the 4 groups and filtered through a 70 *μ*m cell strainer cap (Corning Falcon, USA) into a fresh 5 ml polypropylene tube [20]. To isolate native BMSC subsets from mouse BM cells, up to 10^6^ cells were incubated with 20 *μ*L of fluorescein isothiocyanate (FITC)-conjugated CD45 (Biolegend, CA, USA) and 20 *μ*L phycoerythrin (PE)-conjugated CD44 (Biolegend, CA, USA) for 20 min at room temperature avoiding light, washed in FACS buffer, and centrifuged (5 min at 500×*g*) [21]. Sorting gates were created by selecting CD45-CD44^+^ cells using a BD FACS Aria II (BD Biosciences) with a 100 *μ*m nozzle (Figure 1). Sort-purified BMSCs were collected into PBS containing 30% FBS and centrifuged (15 min at 1000×*g*). Mice sort-purified BMSCs were collected and stored at −80°C for further analysis.

### 2.6. Peripheral Blood Counts in Mice

The anticoagulant blood samples were collected from the orbital vein of mice. White blood cells (WBC), red blood cells (RBC), hemoglobin (Hb) concentration, and platelets (PLT) were measured by an automatic modular animal blood and humoral analyzer (Sysmex XN-2000V, Shanghai, China) after the interventions of BSJPQYF or saline solution for 14 days.

### 2.7. Enzyme-Linked Immunosorbent Assay (ELISA)

The blood samples were collected from the orbital vein of mice and allowed to stand for 1 h at room temperature, then the serum was collected by centrifugation (15 min at 5000 × rpm). The concentrations of TNF-*α*, TGF-*β*, and IFN-*γ* in serum were measured with the ELISA kit (Multi Sciences Biotech, Zhejiang Province, China). The procedure was strictly in accordance with the kit instructions and the required indicators were measured in a microplate reader (PerkinElmer, EnSpire, MA, the United States) [22].

### 2.8. Western Blot Analysis

Total proteins from sort-purified BMSCs were extracted by a RIPA buffer supplemented with 1% of PMSF and 1% phosphatase inhibitor cocktail following the standard protocol, then protein concentrations were determined using a bicinchoninic acid protein assay kit (Epizyme Biomedical Technology, Shanghai, China). The protein (20 *μ*g/well) extracts in equal amount were separated by SDS-PAGE (10%) and electro-transferred to a 0.2 *μ*m PVDF membrane. The membranes were blocked with 5% (w/v) bovine serum albumin for 1 h at room temperature and incubated with primary antibodies against mouse PI3K (Cell Signaling Technology, cat: 4249, 1 : 1000), AKT (Cell Signaling Technology, cat: 4691, 1 : 1000), p-AKT (Cell Signaling Technology, cat: 4060, 1 : 1000), NF-*κ*B (Cell Signaling Technology, cat: 8242, 1 : 1000), p-NF-*κ*B (Cell Signaling Technology, cat: 3033, 1 : 1000), cleaved caspase-3 (Cell Signaling Technology, cat: 9664, 1 : 1000), TNF-*α* (Cell Signaling Technology, cat: 3707, 1 : 1000), and GAPDH (Cell Signaling Technology, cat: 5174, 1 : 1000) at 4°C for 20 h. HRP-conjugated anti-rabbit IgG (Cell Signaling Technology, cat: 7074, 1 : 5000) were applied to membranes for 1 h at room temperature. The membranes were visualized with ECL Substrate (Bio-Rad, CA, the United States). Target proteins were normalized to GAPDH and quantified with Image Lab software (Bio-Rad, CA, the United States).

### 2.9. Statistical Analysis

The *t*-test and one-way (ANOVA) analysis of variance were used to analyze the significance of the differences between the groups by SPSS 19.0 (IBM, Armonk, NY, USA). All data in the text were expressed as mean ± standard deviation. Values of *P* < 0.05 denote statistically significant differences between groups.

## 3. Results

### 3.1. Components Identification of the Chemical Constituents of BSJPQYF

**Figure 2:**
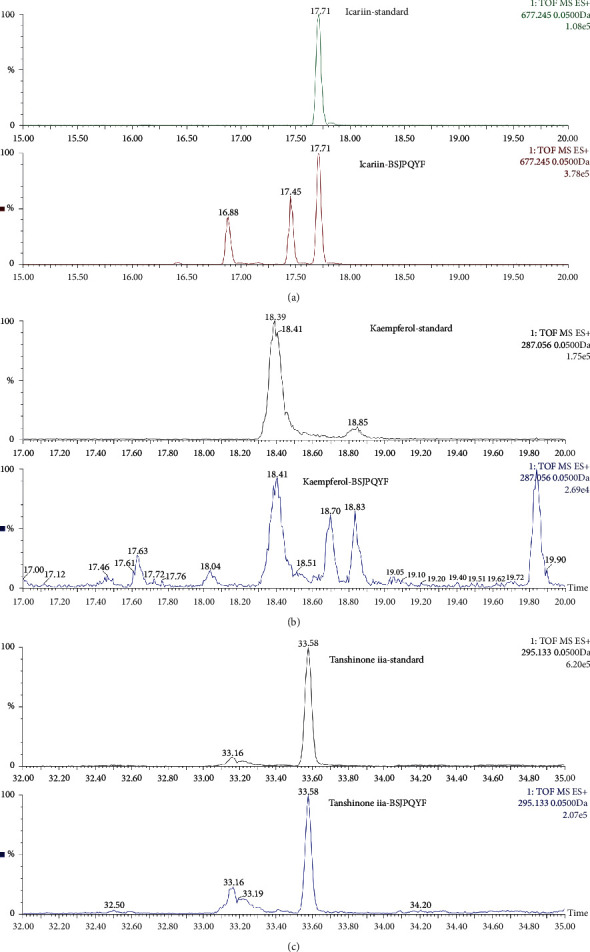
Ion maps of icariin (a), kaempferol (b), and tanshinone iia (c) in BSJPQYF and standards were determined by ESI-MS.

The ESI-MS method was adopted to compare the compounds between the standard and BSJPQYF, and three main compounds were identified. The ion chromatograms of the three standard compounds and BSJPQYF are shown in Figure 2. The retention times of three identified compounds, icariin, kaempferol, and tanshinone iia were 17.71, 18.41, and 33.58 min, respectively.

### 3.2. Effect of BSJPQYF on Peripheral Blood Cell Counts in AA Mice

**Figure 3:**
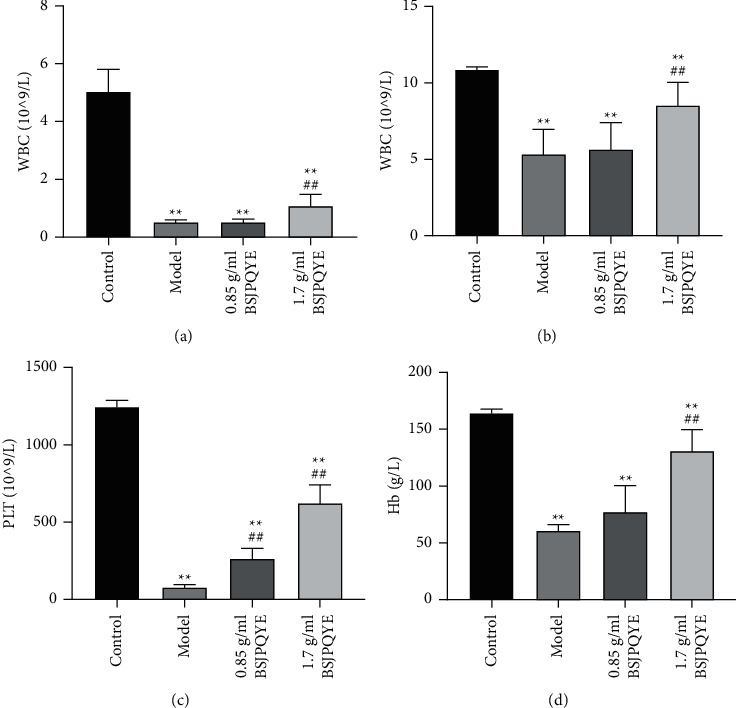
White blood cells (WBC) (a), red blood cells (RBC) (b), platelets (PLT) count (c), and hemoglobin (Hb) concentration (d) in groups of control, model, 0.85 g/mL BSJPQYF, and 1.7 g/mL BSJPQYF. Data are expressed as mean ± SD (*n* = 5). ^*∗∗*^*P* < 0.01 versus the control group; ^##^*P* < 0.01 versus the model group.

As shown in Figure 3, mice in the model group showed pancytopenia with significant reduction on WBC, RBC, PLT counts, and Hb concentrations compared with the control group on day 14 (*P* < 0.01). Compared with the model group, WBC, RBC, PLT counts, and Hb concentrations significantly increased in the 1.7 g/mL group (*P* < 0.01). However, there was no obvious change between the model group and 0.85 g/mL BSJPQYF group on peripheral blood cell counts.

### 3.3. BSJPQYF Alleviated Myelosuppression

**Figure 4:**
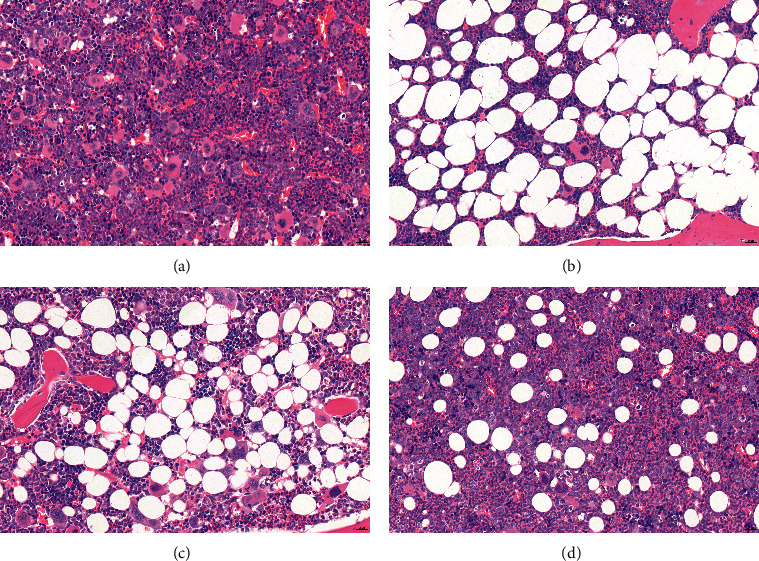
Representative hematoxylin and eosin (HE) stained femur section (original magnification × 40) in groups of control (a), model (b), 0.85 g/mL BSJPQYF (c), and 1.7 g/mL BSJPQYF (d).

As we discovered, TBI and lymphocytes injection induced mice pancytopenia and attacked BM cells. Bone marrow sections of the control group showed abundant BM cells and fewer adipocytes (Figure 4(a)). In contrast, the nucleated cells were replaced by adipose cells largely in the model group (Figure 4(b)). Compared with the model group, we observed that both 0.85 g/mL and 1.7 g/mL BSJPQYF treatment increased the number of BM cells (Figures 4(c) and 4(d)).

### 3.4. Effect of BSJPQYF on the Expression of TNF-*α*, TGF-*β*, and IFN-*γ*

**Figure 5:**
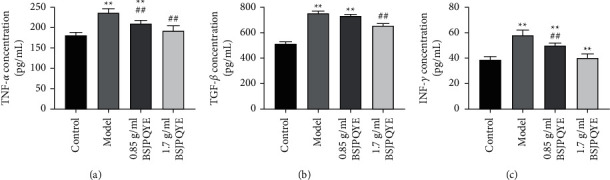
Effect of BSJPQYF on the expression of TNF-*α* (a), TGF-*β* (b), and INF-*γ* (c) in the groups of control, model, 0.85 g/mL BSJPQYF, and 1.7 g/mL BSJPQYF. Data are expressed as mean ± SD (*n* = 5). ^*∗∗*^*P* < 0.01 versus the control group; ^##^*P* < 0.01 versus the model group.

The levels of TNF-*α*, TGF-*β*, and IFN-*γ* in peripheral blood of each mouse were measured by ELISA assay. As shown in Figures 5(a)–5(c), compared with the control group, the expression level of TNF-*α*, TGF-*β*, and IFN-*γ* significantly increased in the model group (*P* < 0.01). After treatment by BSJPQYF of 1.7 g/mL for 14 days, the expressions level of TNF-*α*, TGF-*β*, and IFN-*γ* significantly decreased compared with the model group (*P* < 0.01) (Figures 5(a)–5(c)). Compared with the model group, in 0.85 g/mL BSJPQYF group, TNF-*α* and IFN-*γ* significantly decreased as well (*P* < 0.01), but there was no significant difference of TGF-*β*. In addition, there was no significant difference of TNF-*α* and IFN-*γ* levels between control group and 1.7 g/mL BSJPQYF group (Figures 5(a) and 5(c)).

### 3.5. Effect of BSJPQYF on Expressions of the PI3K/AKT/NF-*κ*B Signaling Pathway in BMSCs of AA Mice

**Figure 6:**
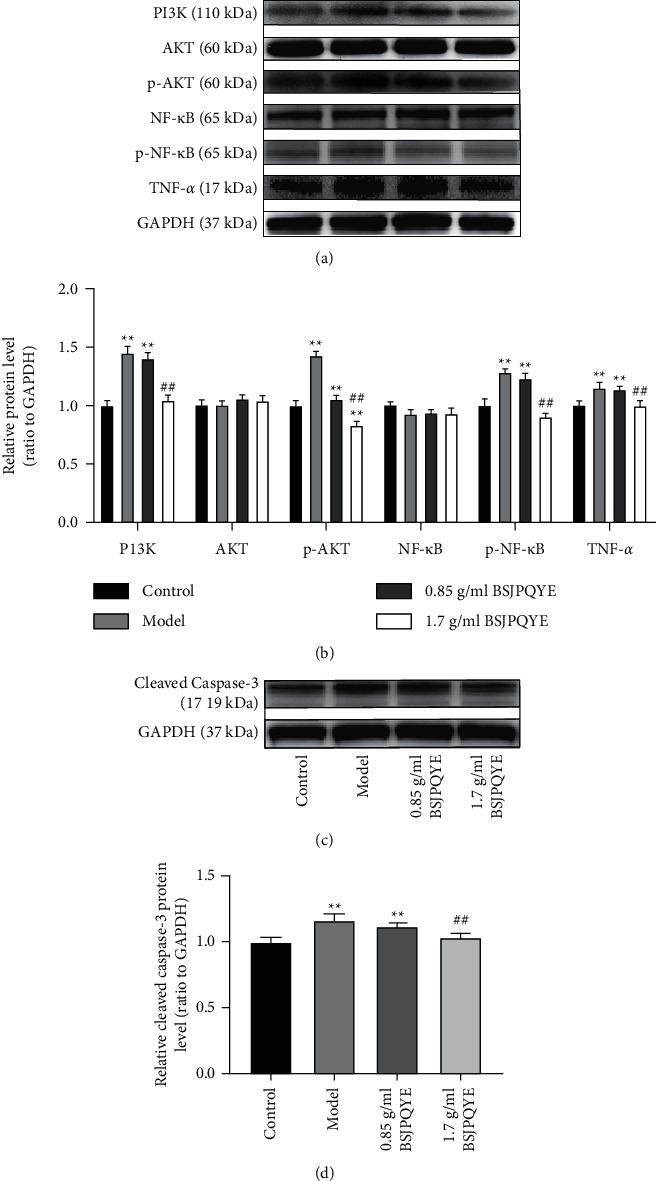
Effect of BSJPQYF on PI3K, AKT, p-AKT, NF-*κ*B, p-NF-*κ*B, TNF-*α*, and cleaved caspase-3 in BMSCs of mice. Groups from left to right, respectively: control group, model group, low dose (0.85 g/mL BSJPQYF) group, and high dose (1.7 g/mL BSJPQYF) group. (a) Protein bands of PI3K, AKT, p-AKT, NF-*κ*B, p-NF-*κ*B, and TNF-*α*. (b) Quantitative analysis of PI3K, AKT, p-AKT, NF-*κ*B, p-NF-*κ*B, and TNF-*α* expression alteration in BMSCs of mice. (c) Protein band of cleaved caspase-3. (d) Quantitative analysis of cleaved caspase-3 expression alteration in BMSCs of mice. Data are expressed as mean ± standard deviation (*n* = 3). ^*∗∗*^*P* < 0.01 versus the control group; ^##^*P* < 0.01 versus the model group.

The PI3K/AKT/NF-*κ*B signaling pathway plays an important role in regulating the inflammatory activity [23]. We collected BMSCs by fluorescence-activated cell sorting (FACS) and performed western blot experiment to assess the expression levels of PI3K/AKT/NF-*κ*B signaling pathway. As shown in Figure 6(a), the protein expressions of PI3K, p-AKT, and p-NF-*κ*B in the model group were significantly higher than those in the control group (*P* < 0.01), but reversed after administration of BSJPQYF. Moreover, the protein expressions of p-AKT and p-NF-*κ*B in the 1.7 g/mL BSJPQYF group were even lower than that in the control group. As expected, the protein expressions of AKT and NF-*κ*B had no statistical difference (Figure 6(b)).

To further understand the role of TNF-*α* molecule expressed by BMSCs, we performed western blot to evaluate TNF-*α* expression levels in BMSCs collected from four groups. As shown in Figure 6(a), the protein expression change trend of TNF-*α* in four groups was consistent with PI3K, p-AKT, and p-NF-*κ*B.

### 3.6. Effect of BSJPQYF on Expressions of Cleaved Caspase-3 in BMSCs

To further explore whether myelosuppression in AA mice was associated with apoptosis, we evaluated the cleaved caspase-3 expression level in BMSCs collected from four groups as well. As shown in Figure 6(c), compared with the control group, the expression of cleaved caspase-3 in the model group was significantly increased (*P* < 0.01), while that in the 1.7 g/mL BSJPQYF group was decreased (*P* < 0.01).

## 4. Discussion

BSJPQYF is a complex system in which the active ingredients such as icariin, kaempferol, and tanshinone iia were identified by ESI-MS (Figure 1). Icariin has been shown to promote the migration, proliferation, osteogenic, and chondrogenic differentiation on BMSCs [24–26]. Besides, *in vivo* study showed the iron overload which led to BMSC dysfunction could be suppressed by icariin, which might be related to the activation of PI3K/AKT/mTOR pathway and the inhibition of ERK1/2 and JNK pathways [27]. Kaempferol, a natural flavonoid, possesses potent anti-inflammatory and antioxidant functions [28, 29] which could inhibit BMSC adipogenesis by promoting lipid catabolism, reducing anabolism, and lipid accumulation *in vitro* and *in vivo* [30, 31]. In addition, it has been reported that tanshinone iia, the main component of *Salvia miltiorrhiza*, works by reducing the production of inflammatory mediators and restoring abnormal signaling pathways. For example, it exerts anti-inflammatory effects through the TLR/NF-*κ*B pathway and the MAPKs/NF-*κ*B pathway [32], as reported. Besides, a study showed that tanshinone iia has strong anti-lipogenic effects [33].

In this research, we successfully constructed the immune-mediated AA mouse model to verify the effectiveness of BSJPQYF *in vivo*. The results showed that BSJPQYF dose-dependently and effectively increased the WBC, RBC, PLT counts, and Hb concentrations in peripheral blood. A histopathological experiment of femurs revealed that BSJPQYF alleviated myelosuppression in mice. In the 100 mg/mL BSJPQYF-treated mice, the bone histopathological sections showed increased bone marrow cells, which were similar to that in the control group. Moreover, there was largely reduction of adipose cells compared with the model group.

It has been demonstrated that levels of IFN-*γ* and TNF-*α* were increased in the SAA and were declined after effective treatment [34–36]. Meanwhile, TGF-*β*1, described as a regulator of hemopoiesis, could impair lymphoid-biase hematopoietic stem cell (ly-HSC) proliferation *in vitro* at lower levels [37, 38]. These were consistent with our experimental results. Our results indicated that BSJPQYF at high dose (1.7 g/mL) significantly reduced TNF-*α*, TGF-*β*, and IFN-*γ* expression level, which were close to that in the control group.

BMSCs, the most important components of BM microenvironment for hematopoiesis, support the long-term maintenance and differentiation of HSC, and exert the immunomodulatory effects to provide an immunoprotected microenvironment [14, 39, 40]. Higher concentrations of IL-6, IFN-*γ* and TNF-*α*, and IL-1*β* were found in the conditioned medium of SAA BMSCs *in vitro* [41]. In the present study, a high expression of PI3K, p-AKT, p-NF-*κ*B, and TNF-*α* protein was observed in BMSCs from AA mice. However, these proteins were decreased in the BSJPQYF treatment group (Figure 6(a)). Therefore, we suggested that TNF-*α* expressed by BMSCs may contribute to the high TNF-*α* level in AA mice, which might be inhibited by BSJPQYF *via* the PI3K/Akt/NF-*κ*B signaling pathway. Interestingly, the expression of p-AKT and p-NF-*κ*B protein in the 1.7 g/mL BSJPQYF group was even lower than that in the control group (Figure 6(b)). Consequently, we speculate that BSJPQYF can directly regulate the phosphorylation of AKT and NF-*κ*B, but the precise mechanism and effective compounds need further research and verification.

Previous study revealed that SAA BMSCs were prone to early senescence and apoptosis [41], and BMSCs were induced to apoptosis by TNF-*α* stimulation *in vitro* [42]. Caspase-3, taken as an effector molecule in the process of apoptosis, can mediate the cell disassembly intrinsic to apoptosis and precipitate the DNA fragmentation [43–45]. The caspase-3 zymogen turns into an activated state (cleaved caspase-3) after cleaved by different proteases. Cleaved caspase-3 further cuts different substrates, leading to the expansion of the protease cascade, eventually to cell apoptosis [46]. We assessed the expression of cleaved caspase-3 protein in four groups of BMSCs to determinate whether BMSCs from AA mice were induced to apoptosis. As expected, a higher expression of cleaved caspase-3 was observed in the model group. Nevertheless, the protein expression of cleaved caspase-3 was dramatically decreased in the BSJPQYF treatment group, especially in the 1.7 g/mL BSJPQYF group (Figure 6(c)).

## 5. Conclusion

**Figure 7:**
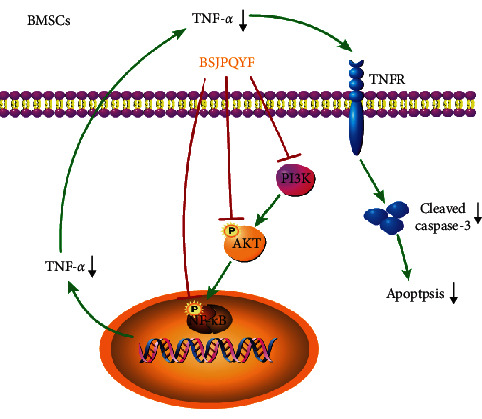
Possible molecular mechanism associated with BSJPQYF treating AA : PI3K/AKT/NF-*κ*B pathway.

Our research data indicated that BSJPQYF could alleviate myelosuppression and promote the recovery of peripheral pancytopenia in AA mice. We speculated that the high expressions of TNF-*α*, TGF-*β*, and IFN-*γ* were related to the pathogenesis of AA, and BSJPQYF played a role in suppressing the expression of TNF-*α via* inhibiting the PI3K/AKT/NF-*κ*B signaling activity, eventually reduced apoptosis in BMSCs (Figure 7). In addition, these therapeutic effects of BSJPQYF might be related to the identified compounds of icariin, kaempferol, and tanshinone iia. In this study, we observed that TNF-*α* production in peripheral blood and BMSCs of AA-mice decreased after treatment with BSJPQYF; however, we are not sure whether there is a direct cause-and-effect relationship. There is a need for further research to make it clear.

## Figures and Tables

**Table 1:** Compositions of Bushen Jianpi Quyu Formula.

Chinese names	Latin name	Weight (g)
Shu Di Huang	*Rehmannia glutinosa*	20
Sheng Di Huang	*Radix rehmanniae*	15
Rou Gui	*Cinnamon*	4
Dan Sheng	*Salvia miltiorrhiza*	10
Dang Gui	*Angelica sinensis*	10
Tu Si Zi	*Cuscuta*	15
Bu Gu Zhi	*Psoralen*	20
Xian Ling Pi	*Herba epimedii*	20
Tai Zi Sheng	*Pseudostellaria heterophylla*	30
Zhi He Shou Wu	*Prepared fleece flower root*	10
Chi Shao	*Peony*	10
Mu Dan Pi	*Cortex moudan*	9
Fu Lin	*Indian Bread*	10
Bai Zhu	*Large head*	12
Shan Yao	*Yam*	30
Gan Cao	*Liquorice*	3

## Data Availability

The data used to support the findings of this study are available from the corresponding author Baodong Ye upon reasonable request.
